# Management of pancreatic pseudocysts—A retrospective analysis

**DOI:** 10.1371/journal.pone.0184374

**Published:** 2017-09-06

**Authors:** Sebastian Rasch, Bärbel Nötzel, Veit Phillip, Tobias Lahmer, Roland M. Schmid, Hana Algül

**Affiliations:** Klinik und Poliklinik für Innere Medizin II, Klinikum rechts der Isar, Technische Universität München, Munich, Germany; Chang Gung Memorial Hospital Kaohsiung Branch, TAIWAN

## Abstract

**Background:**

Pancreatic pseudocysts arise mostly in patients with alcohol induced chronic pancreatitis causing various symptoms and complications. However, data on the optimal management are rare. To address this problem, we analysed patients with pancreatic pseudocysts treated at our clinic retrospectively.

**Methods:**

We searched our clinical database for the diagnosis pancreatitis from 2004 till 2014, selected patients with pseudocysts larger than 10 mm and entered all relevant information in a database for statistical analysis.

**Results:**

In total, 129 patients with pancreatic pseudocysts were treated at our institution during the study period. Most patients suffered from alcohol induced chronic pancreatitis (43.4%; 56/129). Pseudocysts were more frequent in female than in male (2:1) and were mainly located in the pancreatic head (47.3%; 61/129). Local complications like obstructive jaundice were associated with the diameter of the cysts (AUC 0.697 in ROC-curve analysis). However, even cysts up to a diameter of 160 mm can regress spontaneously. Besides a lower re-intervention rate in surgically treated patients, endoscopic, percutaneous and surgical drainage are equally effective. Most treatment related complications occur in large pseudocysts located in the pancreatic head.

**Conclusion:**

Conservative management of large pseudocysts is successful in many patients. Therefore, indication for treatment should be made carefully considering the presence and risk of local complications. Endoscopic and surgical drainage are equally effective.

## Introduction

Pancreatic pseudocysts belong to the diverse entity of pancreatic fluid collections and cystic pancreatic lesions. As features and complications of the different fluid collections and cystic lesions are variable, a classification system was established in 1992 by a consensus meeting in Atlanta and was revised in 2011.[1, 2] According to the revised Atlanta classification, pancreatic pseudocysts usually develop with a delay of at least 4 weeks to the initiating event and are characterized by a well defined inflammatory wall and a homogeneous fluid content without necrosis.[1] Particularly small pseudocysts are difficult to differentiate from cystic tumors of the pancreas.

**Fig 1:**
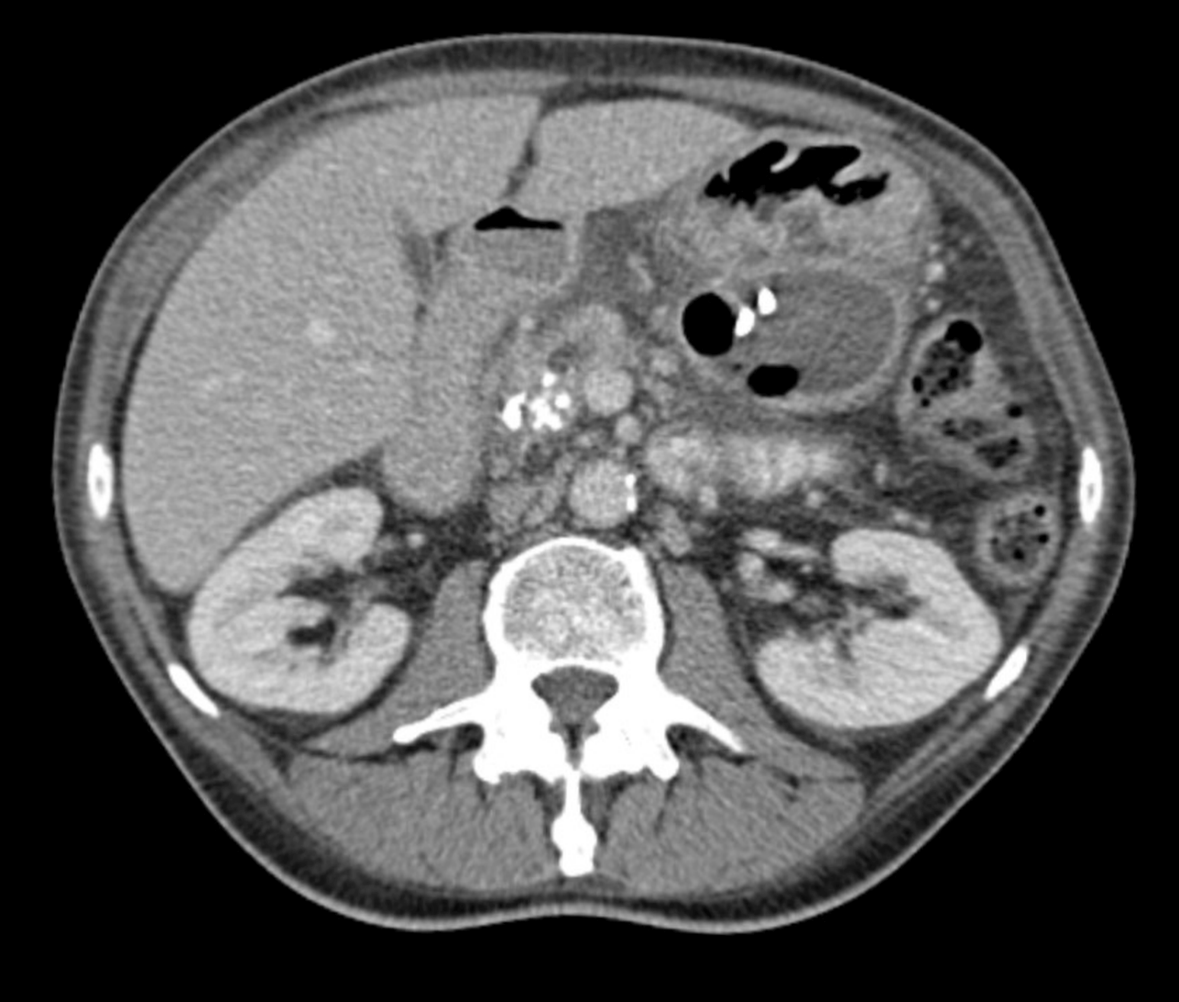
Abdominal CT-scan with venous contrast: Pancreatic pseudocyst with transgastric drainage.

About 70% of pancreatic pseudocysts arise in patients with alcohol induced chronic pancreatitis. In addition, pseudocysts can also evolve after acute pancreatitis, trauma, and surgery.[3–5] Although symptoms and complications are diverse and heavily depend on localisation and size of the pseudocyst, most frequently patients with pancreatic pseudocysts present with abdominal pain.[6] Accordingly, treatment is variable and apart from a few clear indications like infection of the cyst or biliary obstruction neither well defined nor standardized. As the few published studies on pancreatic pseudocysts rely on relatively few patients and do not use uniform definitions, meta-analysis with a sufficient cohort size are difficult so far.[5, 7] Additionally, endosonographic drainage techniques evolved in the last decade and more and more replace surgical drainage procedures like pseudocystojejunostomy—the former standard of therapy. (Fig 1) Although endosonographic drainage has been compared to conventional drainage techniques in randomized controlled trials, in particular the question which patient has to be treated when has hardly been addressed.[7, 8]

With this unicenter retrospective analysis we aimed to address this problem by evaluating treatment indication, analysing treatment options and assessing the course of the disease and the outcome.

## Materials and methods

We performed a unicenter retrospective analysis of patients presenting with pancreatic pseudocysts between 2004 and 2014 at the tertiary referral center Klinikum rechts der Isar der Technischen Universität München. We screened our administrative diagnosis database for patients with pancreatitis (International Classification of Diseases (ICD)-10 code K85 and K86; n = 3281). Pancreatic pseudocysts were defined according to the revised Atlanta classification.[1] Patients with pancreatic pseudocysts larger than 10 mm who presented more than one time were eligible for this study. Patients with cysts suspicious of dysplasia or walled of necrosis were excluded. We reviewed the medical charts of all 3281 patients diagnosed with chronic and acute pancreatitis. Thus, we identified 129 patients with pancreatic pseudocysts that met the inclusion criteria. All patients were registered in a database containing 105 clinical parameters. The study was approved by the local ethics committee (Ethikkommission der Fakultät für Medizin der Technischen Universität München, project number 466/14). Written consent was specifically waived by the approving institutional review board.

Statistical analysis was performed using IBM SPSS Statistics 22 (SPSS Inc, Chicago, Illinois, USA). To compare qualitative parameters, chi-square test was used and in small samples (expected frequency of test variable less than 5), Fisher's exact test was used. For the analysis of quantitative parameters, Mann-Whitney-U test was employed. All statistical tests were two sided with a level of significance (p-value) of 5%. A binary logistic regression model was used to analyse the effect of patient characteristics on the development of symptomatic cysts or local complications. Factors with a p-value below 0.1 in univariate analysis or a high probability according to our data or the literature were included in regression analysis. The p-value remained 0.05 after a Benjamini-Hochberg correction to control the false discovery rate, as several regression analyses were performed.[9] Descriptive data are presented as mean ± standard deviation (SD) for normally distributed parameters and median, range and interquartile range (IQR) for not normally distributed parameters. Risk ratios were displayed as odds ratio (OR) with 95% confidence interval (CI).

### Drainage techniques

All endoscopic drainage procedures were performed under endosonographic guidance by a linear scanner. A gastro- or duodenocystostomy was carried out with a cystostome, fluid specimen were obtained by aspiration and 1–3 double pig tails were placed via a guide wire. For percutaneous drainage pig tail catheters were placed by Seldinger's technique under sonographic or computertomographic guidance. All surgical drainage procedures were cystojejunostomies with a Roux-en-Y reconstruction.

## Results

### Patients`characteristics

Overall, 161 pancreatic pseudocysts in 129 patients were identified. Accordingly, pseudocysts occurred in 3.93% (129/3281) of the patients. Most of the patients`characteristics are summed up in Table 1. The median follow up time was 4.7 months (IQR 1.6–15.1, range 0.2–102). In 6.2% (8/129) of the patients the pseudocyst was an incidental finding, 28.7% (37/129) of the patients were active smokers. Concomitant liver cirrhosis was present in 7.0% (9/129) of the patients, 11.6% (15/129) were suffering from diabetes mellitus type 2 and 5.4% (7/129) of diabetes mellitus type 3c. No pancreatic pseudocyst due to acute pancreatitis was diagnosed after publication of the revised Atlanta classification in 2012.

**Table 1:** Patient characteristics.

**Patient characteristics, n = 129**	
Gender (♂: ♀)	1:2
Mean age (years)	52 (±14.9)
median follow up in days	141 (IQR 48–452; range 3–3056)
**Cyst**	
**Etiology**	
acute pancreatitis	14.7% (19/129)
- alcoholic	42.1% (8/19)
- biliary	57.9% (11/19)
chronic pancreatitis	65.1% (84/129)
*- alcoholic*	*66*.*7% (56/84)*
*- hereditary*	*3*.*6% (3/84)*
*- other/idiopathic*	*29*.*8% (25/84)*
iatrogen and trauma	3.9% (5/129)
Idiopathic pseudocysts	16.3% (21/129)
median number per patient	1 (IQR 1–2; range 1–5)
median diameter (mm)	60 (IQR 32–88; range 10–180)
**Location**	
Head	47.3% (61/129)
Body	27.1% (35/129)
Tail	24.8% (32/129)
Extrapancreatic	25.6% (33/129)
**Symptoms**	
abdominal pain	63.6% (82/129)
nausea/vomitting	19.4% (25/129)
weight loss	18.6% (24/129)
Indigestion	6.2% (6/129)
Bloating	6.2% (6/129)
Jaundice	3.9% (5/129)
**Local complications**	
pancreatic duct obstruction	17.8% (23/129)
blood vessel obstruction	15.5% (20/129)
intestinal obstruction	15.5% (20/129)
bile duct obstruction	13.2% (17/129)
venous thrombosis	8.5% (11/129)
Cholangitis	1.6% (2/129)

**Fig 2:**
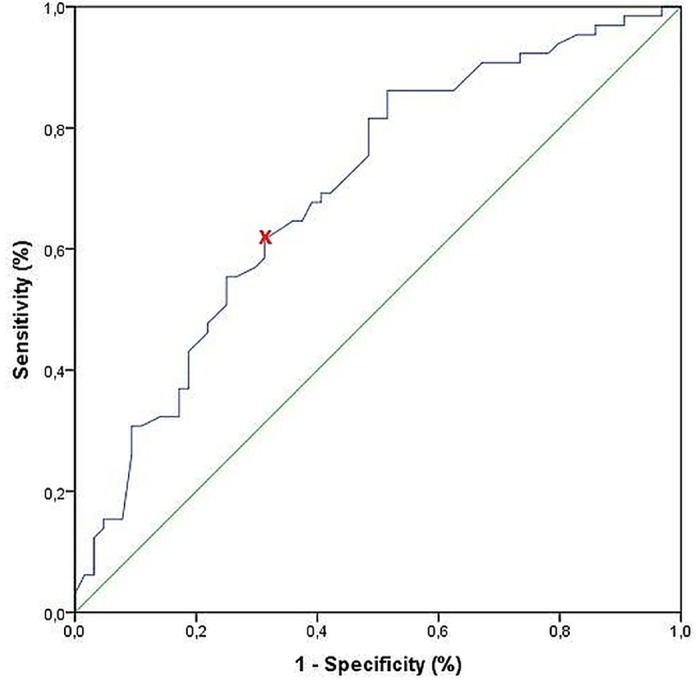
ROC-Curve for cyst diameter as predictor of local complications. Area under the curve (AUC): 0.697. x = cut-off with most equally high sensitivity and specificity: 61,0 mm (sensitivity: 61.5%, specificity: 68.7%).

In multiple logistic regression analysis only cyst number and diameter was associated with a higher rate of local complications like stenosis of the pancreatic or biliary duct, of the intestine or vessels as well as thrombosis or ruptured cyst (number: OR 2.03, CI 1.10–3.76, p = 0.024; diameter: OR 1.30/cm, CI 1.14–1.49, p<0.001). Neither location or the number nor the etiology of the pseudocyst or the gender had an impact on the development of a symptomatic disease or the occurrence of local complications (S1 and S2 Tables). Using receiver operating characteristic (ROC) -curve analysis, cyst diameter is only a fair predictor for the development of local complications. (Fig 2)

### Therapy

An endoscopic or percutaneous puncture of the cyst was performed 54 times. The most frequent indication for puncture was for diagnostic purposes (72.2%, 39/54) to rule out malignancy or if an infection of the cyst was suspected. One puncture was technically unsuccessful, and in one case bleeding occurred after intervention. There was a complete resolution of the cyst after diagnostic puncture in 6 patients. However, repeated intervention was necessary in 50% (27/54) of the cases.

In total, 34.1% (44/129) of the patients were managed conservatively and 65.9% (85/129) required an intervention, respectively. In 40.0% (22/55) the indication for drainage was suspected infection. There was no significant difference in the complication rate after drainage between infected and not infected cysts (9.1% (2/22) versus 24.2% (8/33), P = 0.284). Details on the different treatment options are displayed in Table 2. Patients without an intervention on the cyst had a statistically significant shorter length of hospital stay compared to patients in whom a puncture of the cyst was performed (in days: median 3, IQR 0–13, range 0–30 versus median 14, IQR 9–24, range 0–52, p<0.001). Surgically treated patients had a significantly lower re-intervention rate than patients with percutaneous or endoscopic drainage (0% (0/21) versus 26.5% (13/49), p = 0.007). Apart from that, there were no statistically significant differences considering length of hospital stay, complication rate, and re-intervention rate between the different treatment modalities.

**Table 2:** Comparison of treatment options.

	Conservative Mangement	Endoscopic Drainage	Percutaneous Drainage	Surgical Drainage	Resection
N	44	41	8	6	15
as first intervention	44	68.3% (28/41)	50% (4/8)	83.3% (5/6)	46.7% (7/15)
Median cyst diameter in mm	36,5	84	75	64,5	68
(IQR / range)	(28.5–59.25 / 10–160)	(66.5–100 / 50–180)	(63.5–96.75 / 38–117)	(53.75–109 / 35–160)	(50–100 / 25–154)
Improvement of symptoms	56.8% (25/44)	78% (32/41)	87.5% (7/8)	83.3% (5/6)	80% (12/15)
Decrease of cyst size	66% (29/44)	95.1% (39/44)	87.5% (7/8)	100% (6/6)	100% (15/15)
Complication rate		22% (9/41)	12.5% (1/8)	0% (0/6)	40% (6/15)
most frequent complications		stent occlusion, haemorrhage	haemorrhage		Infection
Reintervention		22% (9/41)	50% (4/8)	0% (0/6)	0% (0/15)
Median hospitalisation in days	3	16	21	19,5	27

The localisation of the pseudocyst had no impact on whether patients were managed conservatively or invasively. Pseudocysts of patients who received an intervention had a significantly higher diameter than those of conservatively managed patients (median 67 mm, IQR 30.75 mm, range 15–180 mm versus median 36.5 mm, IQR 48.5 mm, range 10–160 mm, p<0.001).

Neither cyst diameter, cyst location nor laboratory signs of infection prior to intervention (C-reactive protein > 0.5 mg/dl or white blood cell count > 9 G/l) were associated with complications due to intervention in multivariate logistic regression analysis (S3 and S4 Tables). However, with increasing age, cyst diameter and a location of the cyst in the pancreatic head, there was a higher rate of repeated interventions (age: OR 0.97, CI 0.93–1.00, p = 0.045; cyst diameter: OR 1.22/cm, CI 1.04–1.43, p = 0.012; cyst in pancreatic head: OR 12.81, CI 1.35–121.2., p = 0.026). In total, 16.3% (21/129) of the patients had to be admitted to an intensive care unit including 81% (17/21) of the surgical treated patients in part as routine surveillance after surgery. No patient died as a consequence of treatment for pancreatic pseudocysts.

The occurrence of symptomatic cysts, local complications of the cysts or the cyst diameter did not differ significantly between patients who initially presented to the medical or surgical department. When patients initially presented to the surgical department, significantly more received a surgical drainage or a resection compared to patients who presented to the medical department (42.1% (8/19) versus 4.1% (4/98), p<0.001).

## Discussion

The incidence of pseudocysts after acute pancreatitis is reported to be between 5–16% and after chronic pancreatitis 20–40%, respectively.[10–13] In our patient population pancreatic pseudocysts occurred less frequently in 3.9% after acute and chronic pancreatitis. In 2012 pancreatic pseudocysts were defined in the revised Atlanta classification as 'an encapsulated collection of fluid with a well defined inflammatory wall with minimal or no necrosis' and can since then be clearly distinguished from pancreatic fluid collections and walled of necrosis. According to the revised Atlanta classification, pseudocysts after acute pancreatitis are rare.[1] After the publication of the revised Atlanta classification in 2012 till 2014, no pseudocyst resulting from acute pancreatitis was diagnosed. So the real incidence of pseudocysts after acute pancreatitis is probably at the lower limit of the reported range. Consistent with the literature pancreatic pseudocysts most frequently developed in patients with chronic, alcohol induced pancreatitis.

While the median age of our patients is within the reported range for this patient population, the gender distribution is imbalanced.[14] Over 80% of the patients with chronic pancreatitis are male as the most frequent cause of chronic pancreatitis–alcoholism–is still a predominantly male problem. [15, 16] However, some studies from the USA and the Netherlands describe a tendency towards a more equal gender distribution.[17, 18] Nevertheless, as two thirds of the pancreatic pseudocysts in our patient population arose in female patients, other factors than alcoholism seem to be decisive for pseudocyst development.

The most challenging issue is to find the right patient and the right timepoint for invasive treatment of pancreatic pseudocysts. The indication in patients with local complications like obstructive jaundice is undoubted (Table 3). The rate of local complications correlates with the size of the cysts but not with symptoms like pain, malabsorption or weight loss. In addition, even large cysts can resolve spontaneously. According to the literature, only pseudocysts larger than 50 mm that do not regress within 6 weeks should be treated unless local complications arise. However, this recommendation relies on the first Atlanta consensus conference on acute pancreatitis in 1992.[2, 19, 20]

**Table 3:** Indication for treatment in pseudocysts with local complications.

Immediate treatment is indicated in case of local complications of pancreatic pseudocysts:
- Obstructive Jaundice
- Infection of the pseudocyst
- Obstruction of gastrointestinal tract
- Compression of intestinal vessels
- Hemorrhage

According to our data pancreatic pseudocysts with a size above 60 mm should be treated in case they do not resolve within 6 weeks to avoid the development of local complications. But considering the low sensitivity and specificity of this parameter other aspects like comorbidities and patients`symptoms should be taken into account as well.

According to Ardengh et al. puncture with aspiration and stent placement for endoscopic drainage are equally effective.[21] In contrast our patients frequently required a re-intervention after simple puncture. Consequently simply endoscopic or percutaneous puncture of pancreatic pseudocysts is predominantly a diagnostic procedure and usually drainage placement is required to control symptoms. Endoscopic, percutaneous or surgical drainage as well as resection are effective to improve symptoms and reduce the cyst size. As endosonographic guided punctures are superior to mere gastroscopic punctures all our endoscopic drainages were performed with endosonographic guidance.[22] Recent literature reported similar success and complication rates for surgical and endoscopic interventions, while the length of hospital stay was significantly shorter in endoscopically treated patients.[23–26] Our data are in line with these findings. And yet, surgical therapy seems to be associated with the lowest re-intervention rate. Although in our analysis the sample size is too small to identify significant differences between each intervention, endoscopic drainages tend to result in a shorter hospitalisation and fewer re-interventions than percutaneous drainages which is in line with the literature.[27, 28] So endosonographic drainage should be preferred to percutaneous drainage techniques in pancreatic pseudocysts.

There are conflicting results concerning the outcome of drainage of infected pseudocysts. Sadik et al. reported high complication rates after drainage of infected pseudocysts.[29, 30] Our results support the finding of Varandajuru et al. with low complication rates after drainage of infected pseudocysts.[25] In general, elevated infection parameters, such as C-reactive protein and white blood cell count, were not associated with more complications potentially indicating chronic systemic inflammation with accelerated biological ageing in patients with chronic pancreatitis.[31] Only large pseudocysts located in the pancreatic head are more prone to treatment associated complications like hemorrhage, perforation and formation of fistulae. The complication rate in these large cysts can possibly be further reduced by irrigation via a nasocystic drainage.[32]

## Conclusion

Endoscopic and surgical drainage of pancreatic pseudocysts are equally safe and effective treatment options. Most interestingly, even large pseudocysts can regress spontaneously. Accordingly, many patients can successfully be managed conservatively and pseudocysts should only be treated in the presence of local complications like obstructive jaundice or infection. Large prospective studies are needed to identify clear treatment indications for uncomplicated pseudocysts and define the timepoint of intervention.

## Supporting information

S1 Table Variables included in regression model 1 about 'local complications'. (XLSX)

S2 Table Variables included in regression model 2 about 'symptomatic cysts'. (XLSX)

S3 Table Variables included in regression model 3 about 'complications of interventions'. (XLSX)

S4 Table Variables included in regression model 4 about 're-intervention rate'. (XLSX)

## References

[1] Banks PA, Bollen TL, Dervenis C, Gooszen HG, Johnson CD, Sarr MG, et al Classification of acute pancreatitis—2012: revision of the Atlanta classification and definitions by international consensus. Gut. 2013;62(1):102–11. doi: 10.1136/gutjnl-2012-302779 . 23100216 10.1136/gutjnl-2012-302779

[2] Bradley EL 3rd. A clinically based classification system for acute pancreatitis. Summary of the International Symposium on Acute Pancreatitis, Atlanta, Ga, September 11 through 13, 1992. Arch Surg. 1993;128(5):586–90. . 8489394 10.1001/archsurg.1993.01420170122019

[3] Ammann RW, Akovbiantz A, Largiader F, Schueler G. Course and outcome of chronic pancreatitis. Longitudinal study of a mixed medical-surgical series of 245 patients. Gastroenterology. 1984;86(5 Pt 1):820–8. . 6706066

[4] O'Malley VP, Cannon JP, Postier RG. Pancreatic pseudocysts: cause, therapy, and results. Am J Surg. 1985;150(6):680–2. . 3907380 10.1016/0002-9610(85)90407-6

[5] Cannon JW, Callery MP, Vollmer CM Jr. Diagnosis and management of pancreatic pseudocysts: what is the evidence? J Am Coll Surg. 2009;209(3):385–93. doi: 10.1016/j.jamcollsurg.2009.04.017 . 19717045 10.1016/j.jamcollsurg.2009.04.017

[6] Gumaste VV, Pitchumoni CS. Pancreatic pseudocyst. The Gastroenterologist. 1996;4(1):33–43. Epub 03/01. . 8689144

[7] Gurusamy KS, Pallari E, Hawkins N, Pereira SP, Davidson BR. Management strategies for pancreatic pseudocysts. The Cochrane database of systematic reviews. 2016;4:Cd011392 Epub 04/15. doi: 10.1002/14651858.CD011392.pub2 . 27075711 10.1002/14651858.CD011392.pub2 PMC6457582

[8] Park DH, Lee SS, Moon SH, Choi SY, Jung SW, Seo DW, et al Endoscopic ultrasound-guided versus conventional transmural drainage for pancreatic pseudocysts: a prospective randomized trial. Endoscopy. 2009;41(10):842–8. Epub 10/03. doi: 10.1055/s-0029-1215133 Epub 2009 Oct 1. . 19798610 10.1055/s-0029-1215133

[9] Benjamini Y, Hochberg Y. Controlling the False Discovery Rate: A Practical and Powerful Approach to Multiple Testing. Journal of the Royal Statistical Society Series B (Methodological). 1995;57(1):289–300.

[10] Bradley EL, Gonzalez AC, Clements JL Jr. Acute pancreatic pseudocysts: incidence and implications. Ann Surg. 1976;184(6):734–7. ; PubMed Central PMCID: PMCPMC1345417. 999349 10.1097/00000658-197612000-00013 PMC1345417

[11] London NJ, Neoptolemos JP, Lavelle J, Bailey I, James D. Serial computed tomography scanning in acute pancreatitis: a prospective study. Gut. 1989;30(3):397–403. ; PubMed Central PMCID: PMCPMC1378466. 2651228 10.1136/gut.30.3.397 PMC1378466

[12] Maringhini A, Uomo G, Patti R, Rabitti P, Termini A, Cavallera A, et al Pseudocysts in acute nonalcoholic pancreatitis: incidence and natural history. Dig Dis Sci. 1999;44(8):1669–73. . 10492151 10.1023/a:1026691700511

[13] Barthet M, Bugallo M, Moreira LS, Bastid C, Sastre B, Sahel J. Management of cysts and pseudocysts complicating chronic pancreatitis. A retrospective study of 143 patients. Gastroenterol Clin Biol. 1993;17(4):270–6. . 8339886

[14] Jupp J, Fine D, Johnson CD. The epidemiology and socioeconomic impact of chronic pancreatitis. Best Pract Res Clin Gastroenterol. 2010;24(3):219–31. doi: 10.1016/j.bpg.2010.03.005 . 20510824 10.1016/j.bpg.2010.03.005

[15] Lankisch PG, Assmus C, Maisonneuve P, Lowenfels AB. Epidemiology of pancreatic diseases in Luneburg County. A study in a defined german population. Pancreatology. 2002;2(5):469–77. doi: 10.1159/000064713 . 12378115 10.1159/000064713

[16] Ryu JK, Lee JK, Kim YT, Lee DK, Seo DW, Lee KT, et al Clinical features of chronic pancreatitis in Korea: a multicenter nationwide study. Digestion. 2005;72(4):207–11. doi: 10.1159/000089414 . 16260866 10.1159/000089414

[17] Spanier BW, Dijkgraaf MG, Bruno MJ. Trends and forecasts of hospital admissions for acute and chronic pancreatitis in the Netherlands. Eur J Gastroenterol Hepatol. 2008;20(7):653–8. doi: 10.1097/MEG.0b013e3282f52f83 . 18679068 10.1097/MEG.0b013e3282f52f83

[18] Tao N, Sussman S, Nieto J, Tsukamoto H, Yuan JM. Demographic characteristics of hospitalized patients with alcoholic liver disease and pancreatitis in los angeles county. Alcohol Clin Exp Res. 2003;27(11):1798–804. doi: 10.1097/01.ALC.0000095862.30777.D9 . 14634496 10.1097/01.ALC.0000095862.30777.D9

[19] Aghdassi AA, Mayerle J, Kraft M, Sielenkamper AW, Heidecke CD, Lerch MM. Pancreatic pseudocysts—when and how to treat? HPB: the official journal of the International Hepato Pancreato Biliary Association. 2006;8(6):432–41. doi: 10.1080/13651820600748012 ; PubMed Central PMCID: PMC2020756. 18333098 10.1080/13651820600748012 PMC2020756

[20] Lerch MM, Stier A, Wahnschaffe U, Mayerle J. Pancreatic pseudocysts: observation, endoscopic drainage, or resection? Deutsches Arzteblatt international. 2009;106(38):614–21. doi: 10.3238/arztebl.2009.0614 ; PubMed Central PMCID: PMC2770216. 19890418 10.3238/arztebl.2009.0614 PMC2770216

[21] Ardengh JC, Coelho DE, Coelho JF, de Lima LF, dos Santos JS, Modena JL. Single-step EUS-guided endoscopic treatment for sterile pancreatic collections: a single-center experience. Dig Dis. 2008;26(4):370–6. doi: 10.1159/000177024 . 19188730 10.1159/000177024

[22] Varadarajulu S, Christein JD, Tamhane A, Drelichman ER, Wilcox CM. Prospective randomized trial comparing EUS and EGD for transmural drainage of pancreatic pseudocysts (with videos). Gastrointestinal endoscopy. 2008;68(6):1102–11. Epub 07/22. doi: 10.1016/j.gie.2008.04.028 Epub 2008 Jul 21. . 18640677 10.1016/j.gie.2008.04.028

[23] Johnson MD, Walsh RM, Henderson JM, Brown N, Ponsky J, Dumot J, et al Surgical versus nonsurgical management of pancreatic pseudocysts. Journal of clinical gastroenterology. 2008;43(6):586–90. Epub 12/17. doi: 10.1097/MCG.0b013e31817440be . 19077728 10.1097/MCG.0b013e31817440be

[24] Melman L, Azar R, Beddow K, Brunt LM, Halpin VJ, Eagon JC, et al Primary and overall success rates for clinical outcomes after laparoscopic, endoscopic, and open pancreatic cystgastrostomy for pancreatic pseudocysts. Surgical endoscopy. 2008;23(2):267–71. Epub 11/28. doi: 10.1007/s00464-008-0196-2 Epub 2008 Nov 27. . 19037696 10.1007/s00464-008-0196-2

[25] Varadarajulu S, Bang JY, Sutton BS, Trevino JM, Christein JD, Wilcox CM. Equal efficacy of endoscopic and surgical cystogastrostomy for pancreatic pseudocyst drainage in a randomized trial. Gastroenterology. 2013;145(3):583–90.e1. Epub 06/05. doi: 10.1053/j.gastro.2013.05.046 Epub 2013 May 31. . 23732774 10.1053/j.gastro.2013.05.046

[26] Ruckert F, Lietzmann A, Wilhelm TJ, Sold M, Kahler G, Schneider A. Long-term results after endoscopic drainage of pancreatic pseudocysts: A single-center experience. Pancreatology. 2017;17(4):555–60. doi: 10.1016/j.pan.2017.06.002 . 28606430 10.1016/j.pan.2017.06.002

[27] Akshintala VS, Saxena P, Zaheer A, Rana U, Hutfless SM, Lennon AM, et al A comparative evaluation of outcomes of endoscopic versus percutaneous drainage for symptomatic pancreatic pseudocysts. Gastrointestinal endoscopy. 2013;79(6):921–8; quiz 83.e2, 83.e5. Epub 12/10. doi: 10.1016/j.gie.2013.10.032 Epub 2013 Dec 4. . 24315454 10.1016/j.gie.2013.10.032

[28] Keane MG, Sze SF, Cieplik N, Murray S, Johnson GJ, Webster GJ, et al Endoscopic versus percutaneous drainage of symptomatic pancreatic fluid collections: a 14-year experience from a tertiary hepatobiliary centre. Surgical endoscopy. 2015;30(9):3730–40. Epub 12/18. doi: 10.1007/s00464-015-4668-x . 26675934 10.1007/s00464-015-4668-x PMC4992018

[29] Sadik R, Kalaitzakis E, Thune A, Hansen J, Jonson C. EUS-guided drainage is more successful in pancreatic pseudocysts compared with abscesses. World journal of gastroenterology. 2011;17(4):499–505. Epub 01/29. doi: 10.3748/wjg.v17.i4.499 . 21274380 10.3748/wjg.v17.i4.499 PMC3027017

[30] Varadarajulu S, Bang JY, Phadnis MA, Christein JD, Wilcox CM. Endoscopic transmural drainage of peripancreatic fluid collections: outcomes and predictors of treatment success in 211 consecutive patients. Journal of gastrointestinal surgery: official journal of the Society for Surgery of the Alimentary Tract. 2011;15(11):2080–8. Epub 07/26. doi: 10.1007/s11605-011-1621-8 Epub 2011 Jul 23. . 21786063 10.1007/s11605-011-1621-8

[31] Rasch S, Valantiene I, Mickevicius A, Beer S, Rosendahl J, Charnley RM, et al Chronic pancreatitis: Do serum biomarkers provide an association with an inflammageing phenotype? Pancreatology. 2016;16(5):708–14. doi: 10.1016/j.pan.2016.08.004 . 27554641 10.1016/j.pan.2016.08.004

[32] Yuan H, Qin M, Liu R, Hu S. Single-step versus 2-step management of huge pancreatic pseudocysts: a prospective randomized trial with long-term follow-up. Pancreas. 2015;44(4):570–3. doi: 10.1097/MPA.0000000000000307 . 25875795 10.1097/MPA.0000000000000307

